# Antimicrobial Activity on *Streptococcus mutans* and *Enterococcus faecalis* of *Cyperus articulatus* Ethanolic Extracts

**DOI:** 10.3390/plants13050689

**Published:** 2024-02-29

**Authors:** Daniela Vieira de Castro Macambira, José Sousa de Almeida Júnior, Claudia Fernandes de Magalhães Silveira, Sandra Layse Ferreira Sarrazin, Tânia Mara Pires Moraes, Bruno Alexandre da Silva, Antonio Humberto Hamad Minervino, Waldiney Pires Moraes, Lauro Euclides Soares Barata

**Affiliations:** 1 PhD Program Society, Nature and Development, Federal University of Western Pará (PPGSND-UFOPA), Santarém 68040-255, PA, Brazil; dani_macambira@hotmail.com (D.V.d.C.M.); jsalmeidajr@hotmail.com (J.S.d.A.J.); lauroesbarata@gmail.com (L.E.S.B.); 2 Laboratório de Farmacologia Experimental, Universidade Federal do Oeste do Pará, UFOPA, Santarém 68040-255, PA, Brazil; taniafarma@gmail.com; 3 Departamento de Patologia Clínica, Faculdade de Ciências Médicas UNICAMP, Campinas 13083-894, SP, Brazil; dra.claudiasilveira@gmail.com; 4 Programa de Pós-Graduação em Ciências da Saúde, Instituto de Saúde Coletiva da UFOPA, Santarém 68040-255, PA, Brazil; sandra.sarrazin@ufopa.edu.br (S.L.F.S.); bruno.als@ufopa.edu.br (B.A.d.S.); 5 Laboratory of Animal Health, LARSANA, Federal University of Western Pará, UFOPA, Santarém 68040-255, PA, Brazil

**Keywords:** priprioca, ethanolic extract, antimicrobial activity, rhizomes, toxicity assessment

## Abstract

Oral diseases are one of the biggest public health problems worldwide, caused by opportunistic pathogens such as *Streptococcus mutans* and *Enterococcus faecalis*. *Cyperus articulatus* (priprioca) is a plant conventionally used in traditional medicine in the Amazon region. However, little is known about the possible dentistry-related uses of extracts from the rhizomes and solid waste generated by the extraction of essential oils from this vegetable. This study aimed to investigate the chemical composition of volatile compounds and antimicrobial activity through the Minimum Inhibitory Concentration test (MIC and assessment of the toxicity by Hens Egg Test-Chorion Allantoic Membrane (HET-CAM) of the ethanolic extracts from *Cyperus articulatus* intact rhizomes and solid waste. We identified sesquiterpenes as the main constituents, strong antimicrobial activity of the ethanolic extract of intact rhizomes against *S. mutans* (MIC = 0.29 mg/mL), moderate antimicrobial activity against *E. faecalis* of the extract obtained from the solid waste (MIC = 1.17 mg/mL), and absence of toxicity for both tested extracts. The absence of irritation and the antibacterial activity of the ethanolic extract from *C. articulatus* rhizomes and solid waste reveal its potential for use in the alternative control of bacteria that cause oral infections and may present economic viability as a raw material for dental products.

## 1. Introduction

Oral diseases remain one of the largest public health problems worldwide, impacting over 3.5 billion individuals [1]. In 2010, untreated caries in permanent teeth emerged as the predominant condition, affecting 2.4 billion people [2]. Recent data from Brazil’s national survey (SB Brazil 2010) showed a declining trend in caries prevalence among 12-year-olds and adolescents compared to the 2003 survey. Specifically, for individuals aged 15–19 years, the caries index reduced by 35%, and in 12-year-old children it reduced by 26.2%. Conversely, adults had only a slight (17%) decrease and in elderly the prevalence remained unchanged [3].

The SB Brazil 2010 findings suggest that the country shifted from an average prevalence of caries in 2003 (CPO—index of decayed and filled teeth—between 2.7 and 4.4) to a low prevalence condition in 2010 (CPO index between 1.2 and 2.6), according to WHO’s classification [4]. Nonetheless, regional disparities in oral health observed since the 1986 survey perpetuate marked inequalities, particularly among children and adolescents. The north and northeast regions consistently present the worst indicators of oral health, with a CPO of 3.2 and 2.7, respectively [3]. In addition to having the highest caries prevalence values, the northern region also exhibits the highest proportion of decayed teeth (yet to be treated) compared to filled and missing teeth. This highlights the pronounced dental caries disparities in Brazil, with higher oral health impact in the poorest Brazilian regions [5].

Dental caries is a multifactorial pathology involving a susceptible host, microbial biofilm, and cariogenic diet. Among the microorganisms involved, *Streptococcus mutans* stands out as a pivotal initiator of biofilm formation [6]. This microorganism is known for its high cariogenic potential, acidogenicity, and acid tolerance. When exposed to a diet rich in sugar, it metabolizes carbohydrates and produces acids that demineralize the tooth structure [7]. If left untreated, carious lesions penetrate the dentin and reach the pulp, leading to endodontic infections that cause pulpal and periapical alterations. The etiology of these infections is directly linked to microorganisms, with *Enterococcus faecalis* being particularly prominent [8]. Both *S. mutans* and *E. faecalis* are opportunistic pathogens and belong to the group of microorganisms that exhibit high resistance in the oral cavity.

Oral diseases often result in persistent pain, sepsis, reduced quality of life, missed school days, disruptions in family life, and decreased work productivity [9]. Considering these consequences, it is crucial to prioritize the implementation of preventive measures, as the costs associated with treating oral diseases place a substantial economic burden on families and healthcare systems. The most effective approach to controlling dental biofilms is through proper hygiene practices such as brushing and flossing (mechanical methods). However, this method alone may not be fully effective as it relies on the ability and manual dexterity of individuals to perform quality tooth brushing and interdental cleaning. Therefore, it is essential to combine chemical procedures to control biofilm formation [10].

In 2006, medicinal plants and phytotherapy were incorporated into the Unified Health System through the National Policy on Integrative and Complementary Practices [11]. Despite the regulation of phytotherapy, a lack of knowledge regarding the indications and precautions for the use of medicinal plants persists among health professionals. In dentistry, the utilization of medicinal plants for treating oral pathologies, or systemic diseases with oral manifestations, remains relatively unexplored [12].

The highly biodiverse Amazon region harbors approximately 55,000 plant species. Of these, 10,000 may possess medicinal properties. These plants, steeped in centuries-old cultural practices, offer a rich reservoir for therapeutic exploration, requiring the need for rigorous pharmacological evaluation [13,14]. In general, these natural products may be a source for the development of herbal formulations for the treatment of various illnesses [15,16,17]. One common plant in this region is *Cyperus articulatus* (Cyperaceae), an aromatic plant popularly known as priprioca. In the state of Pará, priprioca essential oil has been utilized as a raw material for the cosmetic industry for over 30 years [18,19].

Beyond cosmetics, traditional medicine has leveraged priprioca’s therapeutic attributes for ailments ranging from migraines to fever. Numerous studies have demonstrated the therapeutic properties of priprioca essential oil, including antimicrobial [20], anticonvulsant [21], antiparasitic [22], antioxidant [23], anti-inflammatory, and sedative [24,25] properties. It has also been shown to exhibit potent bactericidal and fungicidal actions against oral microorganisms, effectively controlling biofilm formation [26].

Like many plants, priprioca has a low essential oil yield. The extraction process from the rhizomes of *C. articulatus* generate solid waste, which is often discarded. However, this by-product harbors bioactive compounds, spotlighting its potential as a sustainable resource [27,28].

To date, no study has reported the antimicrobial activity of ethanolic extracts derived from *C. articulatus* rhizomes and its solid waste on *S. mutans* or any other bacteria commonly associated with oral diseases. Therefore, this study aims to elucidate the chemical composition of priprioca ethanolic extracts, assess their toxicity, and evaluate their antimicrobial efficacy against *S. mutans* and *E. faecalis*, providing insights into potential therapeutic applications.

## 2. Materials and Methods

### 2.1. Ethics Committee Approval

This research was approved by the Animal Ethics Committee of the Federal University of Western Pará (UFOPA) under protocol number 1120220228. All procedures were performed in accordance with the guidelines of the National Council for Ethics in Animal Care (CONCEA) and Law No. 11.794/2008 for the care and use of experimental animals.

### 2.2. Plant Material

*Cyperus articulatus* was grown on a small scale in a rural property (54°43′00.10″ W; 02°37′41.10″ S) in the municipality of Santarém, Pará, Brazil. The botanical material was identified by Dr. Antônio Elielson Sousa da Rocha, and a voucher specimen was deposited under registration number MG—207174 in the herbarium of the Museu Paraense Emilio Goeldi (MPEG) (Belém, Pará, Brazil).

### 2.3. Ethanolic Extract from C. articulatus Rhizomes

Immediately after harvesting from a mature plant, 85 g of rhizomes were crushed and subjected to an 8 h extraction process using 96% ethanol in a Soxhlet apparatus. The resulting extract was then concentrated using a rotary evaporator under reduced pressure at a temperature of 50 °C, until the complete evaporation of the solvent. Finally, the extract was carefully packaged in sterile amber bottles and stored refrigerated at 10 °C.

### 2.4. Ethanolic Extract from the Solid Waste of C. articulatus

To obtain the residual essential oil, 300 g of rhizomes underwent hydrodistillation using a Clevenger apparatus. Following the oil extraction process, 62.75 g of the resulting solid waste was subjected to Soxhlet extraction using the same methodology as described for the rhizome extract. The extract obtained from the solid waste was carefully stored in sterile amber bottles and refrigerated at 10 °C.

### 2.5. GC-MS Analysis of Extracts

The volatile compounds present in the ethanolic extracts were analyzed using an Agilent gas chromatograph model 5977E MSD + 7820A gas chromatography-mass spectrometry (GC-MS) system, equipped with an HP-5MS capillary column (30 m × 0.25 mm × 0.25 μm). The analysis was performed under the following conditions: injector temperature of 270 °C, initial temperature of 60 °C, temperature ramp of 3 °C/min, reaching a final temperature of 240 °C, and maintaining it for 60 min (total run time). Helium gas was used as the carrier gas at a flow rate of 1 mL/min. The selective mass detector operated at 70 eV and a m/z range of 50–400. To enhance the analysis, the extracts underwent a methylation sample derivatization process. The preparation involved the addition of 0.2 g of sample to 5 mL of hexane, the mixture was vortexed for 5 min, centrifuged for 5 min, then the supernatant phase was added with 0.5 μL of methanol and a solution of sodium hydroxide (0.5 L). The identified compounds were compared with electronic libraries of the equipment, including NIST, KAAPI, and FFNSC, according to methodology already described in the literature [27,29].

### 2.6. Antimicrobial Evaluation

#### 2.6.1. Microorganisms and Culture Conditions

Lyophilized reference strains of *S. mutans* (ATCC 25175) and *E. faecalis* (ATCC 29212) were reactivated by culturing them in BHI broth (Brain Heart Infusion, DIFCO) to obtain bacterial suspensions. The *S. mutans* and *E. faecalis* suspensions were incubated under aerobic and anaerobic conditions, respectively, for 48 h at 37 °C. The resulting cultures were then transferred to plates containing BHI agar. Inocula were prepared by directly inoculating colonies into 1 mL of sterile saline solution and adjusting them to a standard of 0.5 on the McFarland scale, corresponding to 1.5 × 10^8^ CFU/mL (NCCLS/CLSI, 2006). The turbidity of the suspension was measured using a turbidimeter (MS Tecnopon Instrumentação, Piracicaba, SP, Brazil) to ensure the desired standard on the McFarland scale.

#### 2.6.2. Determination of the Minimum Inhibitory Concentration (MIC)

The antibacterial activity was assessed using the broth microdilution method as recommended by the National Committee for Clinical Laboratory Standards [28]. The initial concentration of the ethanolic extract from the *C. articulatus* rhizomes and the ethanolic extract from the solid waste of *C. articulatus* was 150 mg/mL, dissolved in propylene glycol as the solvent. Serial dilutions were prepared (1:1 dilution factor) using BHI as the diluent to obtain different concentrations. Concurrently, microbial suspensions were adjusted to a standard of 0.5 on the McFarland scale and further diluted in sterile saline solution (1:10 dilution factor) to achieve a final concentration of 1.5 × 10^4^ CFU/mL. The tests were conducted in 96-well plates, with each well containing 90 µL of the specific extract concentration, 90 µL of sterile BHI, and 10 µL of the microbial inoculum. As a positive control, 0.12% chlorhexidine gluconate (PerioGard, Colgate, Campo, SP, Brazil) was used at an initial concentration of 0.6 mg/mL. The viability of the strains, sterility of the medium, and solvent activity was simultaneously monitored. The microplates were incubated in a bacteriological oven at a controlled temperature of 37 ± 2 °C for 24 h. After the incubation period, the inhibition of microbial growth was assessed by adding a 20 µL aqueous solution of resazurin dye (Sigma-Merck, Darmstadt, Germany) (0.02% *w*/*v*), followed by an additional 3 h incubation. MIC, defined as the lowest concentration capable of inhibiting microbial growth, was determined by the presence of a persistent blue color in the wells. A visible color change from blue to pink (indicating the reduction of resazurin to rephazurin) indicated the growth of viable cells. The experiments were performed in triplicate.

### 2.7. Toxicity Assessment: Hens Egg Test-Chorion Allantoic Membrane (HET-CAM)

The HET-CAM test (Hens Egg Test-Chorion Allantoic Membrane) was performed according to the protocol described by the Interagency Coordinating Committee on the Validation of Alternative Methods (ICCVAM) [30].

The study utilized fertilized chicken eggs (*Gallus gallus*) that were incubated for a period of nine days. This timeframe was chosen because it falls within a stage of development where the embryo has not yet formed nervous tissues and does not possess the ability to perceive pain. Following the incubation period, the shell surrounding the air chamber was carefully removed, revealing a moistened internal membrane soaked in saline solution. This membrane was then delicately extracted to expose the chorioallantoic membrane. Next, 0.3 mL of the test formulations were directly applied to the CAM, and the resulting reactions were observed for a duration of 300 s. The time taken for the occurrence of specific events (endpoints) was carefully recorded, monitored, and filmed in seconds.

The following groups were included in the testing: a negative control of 0.9% NaCl, a solvent control of 1% propylene glycol, a positive control of 0.1 mol/L NaOH, 4.68 mg/mL of ethanolic extract from *C. articulatus* rhizomes, and 4.68 mg/mL of ethanolic extract from solid waste of *C. articulatus*. To ensure consistent and reliable results, reference photographs of all the endpoints were used. The three endpoints that were monitored consisted of hemorrhage (bleeding from vessels), vascular lysis (disintegration of blood vessels), and coagulation (denaturation of intra- and extravascular proteins). The concentration of the plant extracts used in the toxicity test was two times higher than the minimal concentration considered to have antimicrobial activity.

The assessment of the test formulations was based on the occurrence of each of the three HET-CAM endpoints at specific time intervals: 30 s, 2 min, and 5 min. A specific score was assigned to the time at which each event took place, reflecting the occurrence of lysis, bleeding, and clotting. Table 1 provides a summary of this relationship.

The scores were then summed to obtain a single numerical value, indicating the irritation capacity of the tested formulation on a common scale ranging from 0 to 21, as shown in Table 2 [30]. The test was performed in triplicate and with the results obtained, an average (between 0 and 21) was determined, indicated for the irritant and non-irritant categories (Table 2) [30].

### 2.8. Statistical Analysis

The results obtained from the toxicity test (HET-CAM) were expressed as the mean ± standard error of the mean. Analysis of variance (ANOVA) was employed to compare the experimental data, while Tukey’s test was utilized to identify differences at a significance level of *p* < 0.05.

## 3. Results

### 3.1. Analysis of the Chemical Composition of Volatile Compounds in the Extracts

The ethanolic extract from *C. articulatus* solid waste yield was 21%. Oxygenated sesquiterpenes (53.59%) and fat acids (33.58%) were predominant in the extract, followed by a minor number of oxygenated monoterpenes (3.69%). Eight components were identified after derivatization of solid waste ethanolic extract, representing 90.86% of the composition, with mustakone being the primary constituent (17.38%) (Table 3).

The ethanolic extract from *C. articulatus* rhizomes yield was 13%. Oxygenated sesquiterpenes (39.23%) and Sesquiterpene hydrocarbons (18.99%) were predominant in the extract, followed by a substantial amount of fat acids (16.63%) and a minor number of oxygenated monoterpenes (1.36%). Forty-three components were identified after derivatization of rhizomes ethanolic extract, representing 76.21% of the composition, with mustakone being the primary constituent (11.36%), followed by gurjunene (9.32%), cyperotundone (6.84%), corymbolone (3.94%), and Isoaromadendrene epoxide (2.94%) (Table 4).

**Figure 1:**
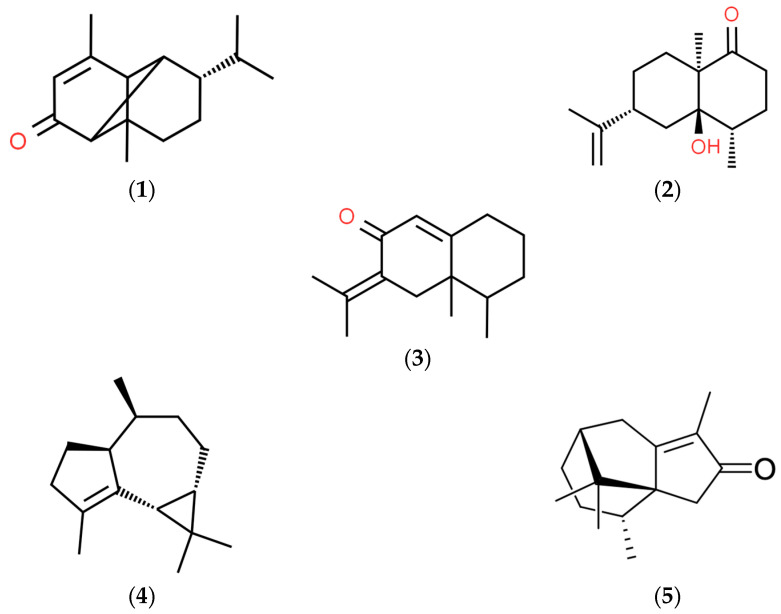
Main volatile from *C. articulatus* ethanolic extracts.

The main volatile constituents identified in the ethanolic extracts from *C. articulatus* (rhizomes and solid waste) were (**1**) mustakone, (**2**) corymbolone, (**3**) dehydrofukinone, (**4**) gurjunene, and (**5**) cyperotundone (Figure 1).

### 3.2. Antimicrobial Evaluation

In our study we have established as having potential antimicrobial activity only the extracts presenting a MIC below 2.0 mg/mL. Therefore, chlorhexidine exhibited antimicrobial activity against both microorganisms tested in lower concentrations (MIC of 0.004 mg/mL against *S. mutans* and 0.002 against *E. faecalis*). When we compare the extracts, we observe that the ethanolic extract from the rhizomes of *C. articulatus* was able to inhibit the growth of *S. mutans* in a lower concentration with values of MIC 0.29 mg/mL; on the other hand, the ethanolic extract of the solid waste showed more expressive MIC values against *E. faecalis*, inhibiting the growth of this bacteria at a concentration of 1.17 mg/mL. Table 5 presents the minimum inhibitory concentrations (MIC), expressed in mg/mL, required to inhibit bacterial growth.

### 3.3. Toxicity Assessment: HET CAM

The analyzed test substances, including the ethanolic extract from *C. articulatus* rhizomes, the ethanolic extract from *C. articulatus* solid waste, and propylene glycol 1%, demonstrated an absence of vascular alterations in the chorioallantoic membrane. This represents the first alterations associated with skin and mucosa irritation and contrasts with the negative control of NaCl 0.9%. Consequently, these substance extracts and solvent did not demonstrate any irritant potential or toxicity and received an “unlabeled” irritation index (II = 0), with no statistical difference when compared to the control group (Na Cl 0.9%). In contrast, sodium hydroxide (NaOH) at 0.1 mol/L was classified as “Serious irritation” due to the appearance of the three endpoints (lysis, hemorrhage, and coagulation) after its contact with the chorioallantoic membrane of embryonated chicken eggs, obtaining an irritation index of 17.67, with statistical difference compared to the control group (*p* ≤ 0.0001) (Table 6).

## 4. Discussion

In this research, we analyzed two plant materials sourced from priprioca (*C. articulatus*). The ethanolic extract from the plant’s rhizomes showcased a phytochemical profile of volatile compounds consistent with findings from other studies in the Amazon region [27,28,29]. Rhizomes serve as a raw material used for extracting priprioca essential oil, which holds substantial commercial value and economic interest, particularly in the perfume industry [18,19]. Numerous studies have highlighted the therapeutic benefits of priprioca essential oil, including antimicrobial, anticonvulsant, antiparasitic, antioxidant, and anti-inflammatory properties, and potent bactericidal and fungicidal activities. Additionally, its effectiveness in controlling oral biofilm formation has been recorded [20,21,26].

Priprioca yields a low essential oil content of approximately 0.45% The processing associated with essential oil extraction results in a substantial volume of plant waste that often goes unused [28]. Preliminary research suggests that this priprioca solid waste contains bioactive compounds with antifungal properties and potential anticancer effects [27,28]. Therefore, in parallel with the evaluation of the plant’s raw materials (rhizomes), we also examined the ethanolic extract obtained from the solid waste generated after extracting the essential oil from *C. articulatus*.

Our results of chemical analyses of volatile compounds in *C. articulatus* extracts align with prior investigations. Corimbolone and mustakone was reported previously as primary sesquiterpenes present in both the rhizome and solid waste extracts derived from *C. articulatus* [22,27,31]. Additionally, cyperotundone, previously detected in the ethanolic extract of the solid waste of *C. articulatus* [27], was found to be an important constituent of the ethanolic extract derived from *C. articulatus* rhizomes [32]. Oleic acid was found in this study to be a major constituent of the ethanolic extract obtained from the solid waste, and it was also identified at a lower concentration in a similar study [29].

The essential oil of *C. articulatus* from Campinas-SP showed the presence of verbenol, myrtenal, myrtenol, verbenone, and e-pinocarveol, at concentrations of 2.10%, 8.16%, 4.61%, 19.57%, and 17.44%, respectively [33]. As expected, these compounds were also found in lower relative concentrations in the ethanolic extract from *C. articulatus* rhizomes, used in this study, at concentrations of 0.42%, 0.11%, 0.27%, 0.40%, and 0.17%, respectively. In the same way, the compounds alpha-copaene (2.2–3.3%), cyperene (1.8–4.3%), cyperadiene (0.3%), trans-calamenene (0.5–0.8%), caryophyllene oxide (4.6–10.8%), isogermacrene D (0.9–1%) and mustakone (9.8–14.5%) identified in the essential oil of *C. articulatus* cultivated in river and coastal areas of the state of Pará in previous research [19] were also found in the ethanolic extract of the rhizome of *C. articulatus* in this study in lower relative concentrations: 0.13%, 0.12%, 0.71%, 0.17%, 2.34, 1.09%, and 11.36%, respectively.

Our results agree with previous research, in which *C. articulatus* oil analyzed by GC-MS showed the presence of alpha-guaiene, β-selinene [26], alpha-calacorene, pogostol, cyperotundone, spathulenol, and thujopsenal [34]. This is similar to our findings, where those compounds were found as constituents of the ethanolic extract of *C. articulatus* rhizome.

Previous reports regarding ethanolic extract from solid waste of *C. articulatus* from Santarém, PA, showed corimbolone as the main compound found (14.25%) [29]. This compound was also present in minor relative amounts within the rhizome ethanolic extract used in this study (3.94%). 

One limitation of the present study was the use of GC-MS analysis for ethanolic extracts. GC-MS is a technique that relies on separating substances from a sample based on their distribution between the stationary (solid or liquid) and mobile (gas) phases. It is most commonly employed for the analysis of volatile gases or substances. Although an important portion of the volatile compounds are removed via distillation of the essential oil, there are still a considerable amount of volatile compounds present in the ethanolic extract, mainly certain volatile sesquiterpenes, that can be detected using GC-MS [27,29].

*S. mutans* and *E. faecalis* are microorganisms found in the oral cavity that are closely linked to the development of dental caries [6] and endodontic infections [8]. To the best of our knowledge, no studies have yet demonstrated the effects of *C. articulatus* extracts on bacteria associated with oral diseases.

Studies have indicated that the chemical compounds α-pinene and β-pinene, present in the essential oil of *C. articulatus*, exhibit antimicrobial activity against gram-positive bacteria and yeasts. These compounds have the potential to impact the viability and/or matrix of the oral biofilm [35]. In our study α-pinene and β-pinene were not found in extracts, suggesting the antimicrobial activity was due to the presence of other chemical compounds. Furthermore, previous research showed strong antimicrobial activity in the essential oil from *C. articulatus* against the oral pathogens *Candida albicans*, *Fusobacterium nucleatum*, *Porphyromonas gingivalis*, *Streptococcus sanguis*, and *Streptococcus mitis*, and chemical analysis of the oil revealed the presence of the sesquiterpene mustakone as the main component, with the presence of alpha-copaene, alpha-guaiene, and β-selinene compounds also recorded in the ethanolic extract from *C. articulatus* rhizomes used in this study [26].

Preliminary studies evaluating the *C. articulatus* extract also identified high concentrations of corimbolone and mustakone and showed in vitro antiplasmodial activity of these substances [29,31]. Similarly, linoleic acid–found in this study to be one of the major compounds in the ethanolic extract of intact rhizomes of *C. articulatus*–was isolated and associated with the death of worms and microfilariae of the bovine parasite *Onchocerca ochengi* [22]. 

Citronellal, a representative acyclic monoterpene compound, showed antibacterial activity against *E. faecalis* (ATCC 29212) in a previous study [36]. The presence of this compound present in the ethanolic extract from *C. articulatus* solid waste may be an explanation for the results found in this study, where the MIC of *E. faecalis* decreased in waste materials compared to plant roots.

The antimicrobial activity of essential oils is not fully understood, but it is thought to be due to the complex mix of components in oils, and not the result of the influence of a single chemical. It has been suggested that the combination of components within the essential oils have either a synergistic or antagonistic relationship, which may cause differences in their antimicrobial properties [36]. Thus, the mechanism of antimicrobial action of the ethanolic extracts from *C. articulatus* rhizomes and solid waste tested in this study on the bacteria that cause oral infections may be related to the synergism of these chemical compounds.

Our research is one of the first reports to investigate the antimicrobial effect of *C. articulatus* extracts against oral bacteria. The strength of the antimicrobial activity of a plant material can be determined based on the MIC values, which corresponds to the lowest concentration of an antibiotic necessary to inhibit the growth of a specific microorganism, essential for developing effective antimicrobial therapies [33]. When MIC values are up to 0.5 mg/mL, the antimicrobial activity is considered strong, while values between 0.6 and 1.5 mg/mL indicate moderate activity [33,37]. Thus, both the ethanolic extract from *C. articulatus* rhizomes and chlorhexidine (positive control) demonstrated strong antimicrobial activity against *S. mutans*, with MIC values below 0.5 mg/mL. Additionally, the ethanolic extract from *C. articulatus* solid waste displayed moderate antimicrobial activity against *E*. *faecalis*, with a MIC value of 1.17 mg/mL [28,37]. 

Mustakone, the main compound of the essential oil from *C. articulatus*, was also identified in the ethanolic extract of the rhizomes, as well as in the ethanolic extract obtained from solid waste derived from oil extraction. However, there were variations in the percentages [22,27,31]. Other studies have identified saponins, flavonoids, terpenes, tannins, and sugars in the extract from the decoction of priprioca rhizomes [25]. The presence of these bioactive substances may be related to the antimicrobial activity observed in this study against *S*. *mutans* and *E*. *faecalis*.

The ethanolic extracts from both the rhizomes and the solid waste from *C. articulatus* did not present toxic activity. In a previous study using the MTT test, the ethanolic extract from *C. articulatus* solid waste did not demonstrate cytotoxic activity in macrophages at concentrations of 12.5, 25, and 50 mg/mL [27]. However, no previous studies have assessed the toxicity of *C. articulatus* extracts using the method employed in this research (HET-CAM). 

Given the absence of toxicity, clinical applications of the ethanolic extract of *C. articulatus* rhizomes and ethanolic extract of *C. articulatus* solid waste at a concentration of 4.8 mg/mL are possible and not risky. 

## 5. Conclusions

Our study demonstrated that the ethanolic extract from *C. articulatus* rhizomes and its solid waste contained sesquiterpenes, with mustakone being the primary constituent. The ethanolic extract from rhizomes had strong antimicrobial activity against *S*. *mutans*, while the extract from the solid waste presented moderate antimicrobial activity against *E*. *faecalis*. The ethanolic extracts from both the rhizomes and the solid waste of *C. articulatus* were safe; they did not present toxic activity. Our results suggest that the Amazon plant priprioca (*C. articulatus*) has potential in the development of dental products against oral infection. However, further studies using drug-resistant bacteria and more complete antimicrobial and cytotoxicity profiles using priprioca purified chemical compounds are necessary.

## Figures and Tables

**Table 1:** Scoring criteria for irritation test with the HET-CAM.

Effects		Points	
	0.5 min (30 s)	2 min (120 s)	5 min (300 s)
**Lysis**	5	3	1
**Bleeding**	7	5	3
**Coagulation**	9	7	5

HET-CAM: Hens Egg test Chorion Allantoic Membrane. Test according to the ICCVAM protocol [30].

**Table 2:** Irritation category according to the HET-CAM score range.

Scoring Range	Irritation Category
0 to 0.9	not labeled
1 to 4.9	slight irritation
5 to 8.9	moderate irritation
9 to 21	severe irritation

HET-CAM: Hens Egg test Chorion Allantoic Membrane. Test according to the ICCVAM protocol [30].

**Table 3:** Constituents identified in the ethanolic extract from *C. articulatus* solid waste.by GC-MS and relative amounts.

Constituents	RT	%
Citronellal	12.47	3.69
Mustakone	33.47	17.38
Rotundone	34.60	6.63
Dehydrofukinone	36.03	15.31
Corymbolone	40.82	14.27
Palmitic acid	42.05	11.04
Oleic acid	47.47	18.26
Stearic acid	48.28	4.28
Oxygenated monoterpenes		3.69
Oxygenated sesquiterpenes		53.59
Fat acids		33.58
Others unidentified		6.41
Total (%)		97.27

RT: retention time per minute; %: relative amount of constituents in the extract fraction.

**Table 4:** Constituents identified in the ethanolic extract from *C. articulatus* rhizomes by GC-MS and relative amounts.

Constituents	RT	%
(E)-Pinocarveol	12.02	0.17
Verbenol	12.29	0.42
(1R)-(-)-Myrtenal	14.22	0.11
Myrtenol	14.44	0.27
Verbenone	14.77	0.40
alpha-copaene	21.64	0.13
Cyperene	22.57	0.12
Cyperadiene	22.86	0.71
alpha.-Guaiene	26.11	0.53
trans-Calamenene	27.62	0.17
9,10-dehydro-Isolongifolene	28.06	0.20
alpha-calacorene	28.39	1.03
Caryophyllene oxide	29.90	2.34
Ledene oxide-(II)	30.18	1.29
Calarene epoxide	31.18	0.25
β-Atlantol	31.36	0.78
Tumerone	31.65	0.93
Valencene	32.17	0.33
Pogostol	32.68	2.30
β-Selinene	32.79	1.71
9-epi-(E)-Caryophyllene	32.89	1.54
Alloaromadendrene	33.08	0.18
Cedrol	33.19	0.59
Isogermacrene D	33.29	1.09
Mustakone	33.50	11.36
Isoaromadendrene epoxide	33.75	2.94
Cyperotundone	34.04	6.84
Eremophilene	34.91	1.02
(-)-Spathulenol	35.05	0.96
Myristic acid	35.22	1.51
Gurjunene	36.06	9.32
Premnaspirodiene	36.31	0.44
cis-Z-.alpha.-Bisabolene epoxide	36.41	0.52
Anhydro-.beta.-rotunol	36.80	1.91
2-epi-(E)-β-Caryophyllene	36.99	1.50
Thujopsenal	37.41	0.23
trans-Z-.alpha.-Bisabolene epoxide	38.03	0.65
Cyperadione	38.90	0.39
Corymbolone	40.84	3.94
Palmitic acid	42.05	4.75
Linoleic acid	47.27	2.43
Oleic acid	47.47	6.71
Stearic acid	48.28	1.24
Oxygenated monoterpenes		1.36
Sesquiterpene hydrocarbons		18.99
Oxygenated sesquiterpenes		39.23
Fat acids		16.63
Others unidentified		20.11
Total (%)		96.32

RT: retention time per minute; %: relative amount of constituents in the extract fraction.

**Table 5:** Minimum Inhibitory Concentration in mg/mL of the ethanolic extract from *C. articulatus* solid waste, ethanolic extract from *C. articulatus* rhizomes and chlorhexidine gluconate against the bacteria *S. mutans* and *E. faecalis*.

Bacterial Strains	Extr EtOH *C. articulatus* Waste	Extr EtOH *C. articulatus* Rizhomes	Chlorhexidine
	MIC (mg/mL)	MIC (mg/mL)	MIC (mg/mL)
*S. mutans* ATCC 25175	>2.0	0.29	0.004
*E. faecalis* ATCC 29212	1.17	>2.0	0.002

Extr EtOH *C. articulatus* waste: ethanolic extract from *C. articulatus* solid waste; Extr EtOH *C. articulatus* rizhomes: ethanolic extract from *C. articulatus* rhizomes; MIC: Minimum Inhibitory Concentration expressed in mg/mL. The initial concentrations of the extract (150 mg/mL) and chlorhexidine (0.6 mg/mL).

**Table 6:** Index and classification of irritation in the chicken egg chorioallantoic membrane test (HET-CAM).

Treatment	II (±SEM)	Irritation Category
(NaOH) 0.1 mol/L (positive control)	17.67 (±1.44) ****	Severe irritation
NaCl 0.9% (negative control)	0 (±0.0)	Not labeled
Propylene glycol 1%	0 (±0.0)	Not labeled
Extr EtOH *C. articulatus* rhizomes (4.68 mg/mL)	0 (±0.0)	Not labeled
Extr EtOH *C. articulatus* waste (4.68 mg/mL)	0 (±0.0)	Not labeled

Extr EtOH *C. articulatus* rhizomes (ethanolic extract from *C. articulatus* rhizomes); Extr EtOH *C. articulatus* waste (ethanolic extract from *C. articulatus* solid waste); II (irritation index); **** Statistical difference compared to the control group NaCl 0.9% (*p* ≤ 0.0001). Results are expressed as the mean (±standard error of the mean).

## Data Availability

All data, tables, and figures in this manuscript are original. Raw data of this experiment is fully evaluable upon request to the corresponding author.

## References

[1] Watt R.G., Daly B., Allison P., Macpherson L.M.D., Venturelli R., Listl S., Weyant R.J., Mathur M.R., Guarnizo-Herreño C.C., Celeste R.K. (2019). Ending the Neglect of Global Oral Health: Time for Radical Action. Lancet.

[2] Kassebaum N.J., Bernabé E., Dahiya M., Bhandari B., Murray C.J.L., Marcenes W. (2015). Global Burden of Untreated Caries: A Systematic Review and Metaregression. J. Dent. Res..

[3] Brasil S.B. Pesquisa Nacional de Saúde Bucal: Resultados Principais/Ministério Da Saúde. Secretaria de Atenção à Saúde. Secretaria de Vigilância Em Saúde. Brasília: Ministério da Saúde 2012, 116. https://bvsms.saude.gov.br/bvs/publicacoes/pesquisa_nacional_saude_bucal.pdf.

[4] Petersen P.E. (2003). The World Oral Health Report 2003: Continuous Improvement of Oral Health in the 21st Century—The Approach of the WHO Global Oral Health Programme. Community Dent. Oral Epidemiol..

[5] Agnelli P.B. (2016). Variação Do Índice CPOD Do Brasil No Período de 1980 a 2010. Rev. Bras Odontol..

[6] Pitts N., Zero D., March P., Ekstrand K., Weintraub J., Ramos-Gomez F., Tagami J., Twetman S., Tsakos G., Ismail A. (2017). Dental Caries. Nat. Rev. Dis. Primers.

[7] Lobo C.I.V., Rinaldi T.B., Christiano C.M.S., De Sales Leite L., Barbugli P.A., Klein M.I. (2019). Dual-Species Biofilms of Streptococcus Mutans and Candida Albicans Exhibit More Biomass and Are Mutually Beneficial Compared with Single-Species Biofilms. J. Oral. Microbiol..

[8] Wade W.G. (2013). The Oral Microbiome in Health and Disease. Pharmacol. Res..

[9] Peres M.A., Macpherson L.M.D., Weyant R.J., Daly B., Venturelli R., Mathur M.R., Listl S., Celeste R.K., Guarnizo-Herreño C.C., Kearns C. (2019). Oral Diseases: A Global Public Health Challenge. Lancet.

[10] Marsh P.D. (2006). Dental Diseases—Are These Examples of Ecological Catastrophes?. Int. J. Dent. Hyg..

[11] Brasil (2006). Ministério da Saúde. A Fitoterapia No SUS Eo Programa de Pesquisas de Plantas Medicinais Da Central de Medicamentos.

[12] D’ávila A.M.M.N., de Cruz J.H.A., Guênes G.M.T., de Oliveira Filho A.A., dos Anjos R.M. (2021). Interações Medicamentosas: Fitoterápicos Utilizados Na Odontologia e Fármacos de Uso Contínuo Dos Pacientes. Arq. Health Investig..

[13] Pereira L.D.P., Da Silva R.O., Bringel P.H.D.S.F., Da Silva K.E.S., Assreuy A.M.S., Pereira M.G. (2012). Polysaccharide Fractions of Caesalpinia Ferrea Pods: Potential Anti-Inflammatory Usage. J. Ethnopharmacol..

[14] De Oliveira Conde N.C., Pereira M.D.S.V., Bandeira M.F.C.L., Venâncio G.N., De Oliveira G.P., Sampaio F.C. (2015). In Vitro Antimicrobial Activity of Plants of the Amazon on Oral Biofilm Micro-Organisms. Rev. Odonto Cienc..

[15] Gomes M.S., Pereira de Mendonça A.K., Cordeiro T.O., Barbosa M.M. (2020). Uso De Plantas Medicinais Na Odontologia: Uma Revisão Integrativa. Rev. Ciências Saúde Nova Esperança.

[16] Mallavadhani U.V., Chandrashekhar M., Nayak V.L., Ramakrishna S. (2015). Synthesis and Anticancer Activity of Novel Fused Pyrimidine Hybrids of Myrrhanone C, a Bicyclic Triterpene of Commiphora Mukul Gum Resin. Mol. Divers.

[17] Chandrashekhar M., Nayak V.L., Ramakrishna S., Mallavadhani U.V. (2016). Novel Triazole Hybrids of Myrrhanone C, a Natural Polypodane Triterpene: Synthesis, Cytotoxic Activity and Cell Based Studies. Eur. J. Med. Chem..

[18] Rocha A.E.S. (2008). As Espécies Cyperaceae Juss. Conhecidas Como Priprioca. POTIGUARA, RC V.; ZOGHBI, MGB Priprioca: Um recurso aromático do Pará.

[19] Zoghbi M.G.B., Guilhon G.M.S., Andrade E.H.A., Vilhena K.S.S. (2008). Química Das Espécies de Cyperus Conhecidas Como Priprioca. POTIGUARA, RC V.; ZOGHBI, MGB Priprioca: Um Recurso Aromático do Pará.

[20] Oladosu I.A., Usman L.A., Olawore N.O., Atata R.F. (2011). Antibacterial Activity of Rhizomes Essential Oils of Two Types of *Cyperus articulatus* Growing in Nigeria. Adv. Biol. Res..

[21] Bum E.N., Schmutz M., Meyer C., Rakotonirina A., Bopelet M., Portet C., Jeker A., Rakotonirina S.V., Olpe H.R., Herrling P. (2001). Anticonvulsant Properties of the Methanolic Extract of *Cyperus articulatus* (Cyperaceae). J. Ethnopharmacol..

[22] Metuge J.A., Babiaka S.B., Mbah J.A., Ntie-Kang F., Ayimele G.A., Cho-Ngwa F. (2014). Anti-Onchocerca Metabolites from *Cyperus articulatus*: Isolation, in Vitro Activity and in Silico ‘Drug-Likeness’. Nat. Prod. Bioprospect..

[23] Kavaz D., Idris M., Onyebuchi C. (2019). Physiochemical Characterization, Antioxidative, Anticancer Cells Proliferation and Food Pathogens Antibacterial Activity of Chitosan Nanoparticles Loaded with *Cyperus articulatus* Rhizome Essential Oils. Int. J. Biol. Macromol..

[24] Desmarchelier C., Mongelli E., Coussio J., Ciccia G. (1996). Studies on the Cytotoxicity, Antimicrobial and DNA-Binding Activities of Plants Used by the Ese’ejas. J. Ethnopharmacol..

[25] Rakotonirina V.S., Bum E.N., Rakotonirina A., Bopelet M. (2001). Sedative Properties of the Decoction of the Rhizome of *Cyperus articulatus*. Fitoterapia.

[26] Bersan S.M.F., Galvão L.C.C., Goes V.F.F., Sartoratto A., Figueira G.M., Rehder V.L.G., Alencar S.M., Duarte R.M.T., Rosalen P.L., Duarte M.C.T. (2014). Action of Essential Oils from Brazilian Native and Exotic Medicinal Species on Oral Biofilms. BMC Complement. Altern. Med..

[27] da Silva É.B.S., Barata L.E.S., Arevalo M.R., Vieira L.Q., Castro W., Ruiz A.L.T.G., Torre A.D., Castro K.C.F., Sartoratto A., Baratto L.C. (2021). Chemical Composition and Antiproliferative Activity of the Ethanolic Extract of *Cyperus articulatus* L. (Cyperaceae). Plants.

[28] Kasper A.A.M., de Sousa S.F., de San Martin B.S., Sartoratto A., Nunes K.M., de Sousa Júnior J.J.V., da Silva S.K.R., Barata L.E.S. (2020). Aproveitamento Dos Resíduos de Priprioca (*Cyperus articulatus* L.) No Controle Alternativo de Fungos Fitopatogênicos. Rev. Ibero-Am. Ciências Ambient..

[29] Assis F.F.V., Da Silva N.C., Moraes W.P., Barata L.E.S., Minervino A.H.H. (2020). Chemical Composition and in Vitro Antiplasmodial Activity of the Ethanolic Extract of *Cyperus articulatus* Var. Nodosus Residue. Pathogens.

[30] NIH ICCVAM (2010). Test Method Evaluation Report: Current Validation Status of In Vitro Test Methods Proposed for Identifying Eye Injury Hazard Potential of Chemicals and Products.

[31] Rukunga G.M., Muregi F.W., Omar S.A., Gathirwa J.W., Muthaura C.N., Peter M.G., Heydenreich M., Mungai G.M. (2008). Anti-Plasmodial Activity of the Extracts and Two Sesquiterpenes from *Cyperus articulatus*. Fitoterapia.

[32] das Zoghbi M.G.B., Andrade E.H.A., Oliveira J., Carreira L.M.M., Guilhon G.M.S.P. (2006). Yield and Chemical Composition of the Essential Oil of the Stems and Rhizomes of *Cyperus articulatus* L. Cultivated in the State of Pará, Brazil. J. Essent. Oil Res..

[33] Duarte M.C.T., Figueira G.M., Sartoratto A., Rehder V.L.G., Delarmelina C. (2005). Anti-Candida Activity of Brazilian Medicinal Plants. J. Ethnopharmacol..

[34] Silva N.C., Goncalves S.F., de Araújo L.S., Kasper A.A.M., da Fonseca A.L., Sartoratto A., Castro K.C.F., Moraes T.M.P., Baratto L.C., de Varotti F.P. (2019). In Vitro and in Vivo Antimalarial Activity of the Volatile Oil of *Cyperus articulatus* (Cyperaceae). Acta Amazon.

[35] Freires I.A., Bueno-Silva B., Galvão L.C.D.C., Duarte M.C.T., Sartoratto A., Figueira G.M., De Alencar S.M., Rosalen P.L. (2015). The Effect of Essential Oils and Bioactive Fractions on Streptococcus Mutans and Candida Albicans Biofilms: A Confocal Analysis. Evid.-Based Complement. Altern. Med..

[36] Yang Z., He S., Wei Y., Li X., Shan A., Wang J. (2023). Antimicrobial Peptides in Combination with Citronellal Efficiently Kills Multidrug Resistance Bacteria. Phytomedicine.

[37] Ferreira G.L.S., Bezerra L.M.D., Ribeiro I.L.A., Morais Júnior R.C.D., Castro R.D. (2018). Susceptibility of Cariogenic Microorganisms to Phytoconstituents. Braz. J. Biol..

